# Localization of Active Brain Sources From EEG Signals Using Empirical Mode Decomposition: A Comparative Study

**DOI:** 10.3389/fnint.2018.00055

**Published:** 2018-11-02

**Authors:** Pablo Andrés Muñoz-Gutiérrez, Eduardo Giraldo, Maximiliano Bueno-López, Marta Molinas

**Affiliations:** ^1^Electronic Instrumentation Technology, Universidad del Quindío, Armenia, Colombia; ^2^Department of Electrical Engineering, Universidad Tecnológica de Pereira, Pereira, Colombia; ^3^Department of Electrical Engineering, Universidad de La Salle, Bogotá, Colombia; ^4^Department of Engineering Cybernetics, Norwegian University of Science and Technology, Trondheim, Norway

**Keywords:** brain mapping, denoising, EEG signals, frequency detection, empirical mode decomposition

## Abstract

The localization of active brain sources from Electroencephalogram (EEG) is a useful method in clinical applications, such as the study of localized epilepsy, evoked-related-potentials, and attention deficit/hyperactivity disorder. The distributed-source model is a common method to estimate neural activity in the brain. The location and amplitude of each active source are estimated by solving the inverse problem by regularization or using Bayesian methods with spatio-temporal constraints. Frequency and spatio-temporal constraints improve the quality of the reconstructed neural activity. However, separation into frequency bands is beneficial when the relevant information is in specific sub-bands. We improved frequency-band identification and preserved good temporal resolution using EEG pre-processing techniques with good frequency band separation and temporal resolution properties. The identified frequency bands were included as constraints in the solution of the inverse problem by decomposing the EEG signals into frequency bands through various methods that offer good frequency and temporal resolution, such as empirical mode decomposition (EMD) and wavelet transform (WT). We present a comparative analysis of the accuracy of brain-source reconstruction using these techniques. The accuracy of the spatial reconstruction was assessed using the Wasserstein metric for real and simulated signals. We approached the mode-mixing problem, inherent to EMD, by exploring three variants of EMD: masking EMD, Ensemble-EMD (EEMD), and multivariate EMD (MEMD). The results of the spatio-temporal brain source reconstruction using these techniques show that masking EMD and MEMD can largely mitigate the mode-mixing problem and achieve a good spatio-temporal reconstruction of the active sources. Masking EMD and EEMD achieved better reconstruction than standard EMD, Multiple Sparse Priors, or wavelet packet decomposition when EMD was used as a pre-processing tool for the spatial reconstruction (averaged over time) of the brain sources. The spatial resolution obtained using all three EMD variants was substantially better than the use of EMD alone, as the mode-mixing problem was mitigated, particularly with masking EMD and EEMD. These findings encourage further exploration into the use of EMD-based pre-processing, the mode-mixing problem, and its impact on the accuracy of brain source activity reconstruction.

## 1. Introduction

EEG signals are difficult to analyze in the time and frequency domain due to their non-linear and non-stationary nature. However, several important characteristics can be extracted to assist the early detection of various disorders using advanced signal analysis techniques (Subha et al., 2010). The localization of active sources in the brain, using electroencephalography (EEG) signals, is a type of brain imaging that has been used in various applications in neuroscience, e.g., to analyze the mechanisms behind language, cognition, sensory functions, and brain oscillations (Jatoi et al., 2014; López et al., 2014). Despite its low spatial resolution, this non-invasive technique has high temporal resolution and is therefore a useful tool for direct real-time monitoring of spontaneous and evoked brain activity, implying spatio-temporal localization of neuronal activity, which can include both temporal and spatial constraints (some including frequency information) (Friston et al., 2008; Gramfort et al., 2013; Babadi et al., 2014; Castaño-Candamil et al., 2015; Costa et al., 2017).

The wavelet transform (WT) and its variants [discrete wavelet transform (DWT) or wavelet packet decomposition (WPD)] are ideal processing tools to finely identify information in non-stationary signals. They have thus been used to analyze EEG signals, which are non-linear and non-stationary. These methods are frequently applied to filter EEG signals (e.g., denoising) or extract EEG signal features (e.g., for statistical analysis). Recent studies have used WT to perform multi-scale analysis and improve the performance of tomographic reconstruction, Rabbouch and Saâdaoui, 2018. The WT has also been used to remove certain artifacts, e.g., power line, electro-oculogram (EOG), electrocardiogram (ECG), or electromyogram (EMG) noise present in the EEG signal (Geetha and Geethalakshmi, 2011) and the DWT compared to EMD in signal decomposition and adaptive filtering (the medical application was denoising preterm EEG) (Navarro et al., 2015). In general, the WT offers a good capacity for feature extraction from non-stationary signals, but the temporal resolution is sometimes poor. The use of the WT in the spatio-temporal localization of neural activity is scarcely reported in the literature, despite its positive traits in dealing with non-stationary signals.

Korats et al. (2016) applied the WT in EEG source localization, in which a data-driven space-time-frequency (STF) dictionary was used to locate sparse sources with non-stationary smooth time courses; the inverse problem was solved using FOcal Underdetermined System Solver (FOCUSS) and RAP-MUSIC (Equivalent Current Dipole - ECD). The data and sources could be analyzed as sparsity in time if the inverse problem was represented through the WT. Another recent study that reported the use of the WT for brain mapping found that inclusion of a WT-based pre-processing stage enhanced spatial reconstruction in terms of the Wasserstein metric for the multiple sparse priors (MSP) and Iterative Regularization Algorithms (IRA-L1) methods vs. the MSP method using raw data. However, this study only analyzed the spatial reconstruction and the temporal resolution of the sub-bands was diminished due to the down-sampling of each sub-band (Muñoz-Gutiérrez et al., 2018).

Along with the DWT and its variants, EMD, with its different versions (EEMD, MEMD, Masking EMD), is one of the most commonly used methods for time-frequency analysis of non-stationary signals. EMD describes the behavior of non-stationary and nonlinear signals by decomposing them into intrinsic mode functions (IMF) to obtain the instantaneous frequency (IF) of the intrinsic modes (Huang et al., 1998). EMD offers better time resolution than the WT, due to its instantaneous frequency property. Both techniques deal well with non-stationary signals, but when good time resolution is crucial, EMD is better. Another advantage of EMD is that it does not need to be combined with other techniques to perform well and adjustment of the parameters of the algorithm is relatively simple.

On the negative side, EMD is limited by the *Mode Mixing* problem. This problem arises when EMD is applied to a signal that exhibits intermittency and/or involves components with spectral proximity (Deering and Kaiser, 2005; Wu and Huang, 2009; Xue et al., 2016; Fosso and Molinas, 2018). In the studies of Rilling and Flandrin (2008) and Rilling et al. (2003), the authors addressed the issue of mode mixing and defined a set of conditions that must exist between the frequency components of a signal to ensure that they can be recognized as independent modes in the EMD decomposition. The mode-mixing problem has been analyzed in different fields. For example, Xue et al. (2016) presented one application for hydrocarbon detection and Tang et al. (2012) showed an application in mechanical systems. Several groups have reported and attempted to mitigate the mode-mixing problem, which can take different forms, although a formal classification has not been yet reported. In a report of Rilling and Flandrin (2008), a rigorous mathematical analysis shows how EMD behaves in the case of a composite two-component signal, explaining the roots of one type of mode-mixing problem, spectral proximity mode mixing. This study identified the frequency-amplitude region within which good separation can be achieved with EMD and the region in which mode mixing occurs. However, a solution that offers good IMF separation has not been available for signal components that reside within the same octave. Fosso and Molinas (2018) very recently proposed a masking signal-based method to separate spectral components that reside within the same octave. In contrast to the guidelines presented in Deering and Kaiser (2005) for selecting the amplitude and frequency of the masking signal, precise amplitudes and frequencies are defined by the boundary map presented in Fosso and Molinas (2018) to reverse a mode-mixing condition. A substantial effort has been devoted to addressing the mode-mixing problem in applications involving EEG signals, by combining various techniques (Chen et al., 2010; Karagiannis and Constantinou, 2011; Alam and Bhuiyan, 2013; Wei et al., 2013; Gonuguntla et al., 2016; Qing-shan et al., 2017; Fosso and Molinas, 2018); MEMD has been reported to handle well the mode-mixing problem in multi-channel data analysis and is therefore an ideal candidate for application in multi-channel EEG signal analysis (Mandic et al., 2013).

Nonetheless, the mode-mixing problem and its effects on the spatio-temporal reconstruction of brain activity have been scarcely addressed in the literature, even if the EMD method exhibits band separation and temporal properties that can be beneficial for the spatio-temporal reconstruction of brain activity. A recent work (Karema et al., 2016) presents an approach for source localization of EEG data based on combining EEMD with standardized low resolution brain electromagnetic tomography (sLORETA) to solve the inverse problem. Accuracy and robustness of the results indicate that this approach deems highly promising in source localization techniques for EEG data. Indeed, there are some promising results, but there is still substantial room for improving the accuracy of the spatio-temporal localization of source activity using EEG (López et al., 2014; Castaño-Candamil et al., 2015; Giraldo-Suarez et al., 2016; Costa et al., 2017).

Here, we have addressed the influence of the mode-mixing problem on the detection of signal sources from various regions in the brain using information from EEG, decomposed with EMD. After analyzing simulated and real brain signals, we detected two types of concurrent mode mixing: one caused by the presence of signal components residing within the same octave (spectral proximity mode mixing) and the other by the presence of intermittency. This motivated us to test the new method of masking EMD and compare the results obtained with the well-established EEMD and MEMD methods. EMD and masking EMD have different scopes and capabilities. The EEMD method was designed to separate components that are mixed due to the presence of intermittency in the signal, which is the root cause of the “split-mode mixing” problem (Wu and Huang, 2009). The EEMD method cannot separate signals that reside within the same octave (spectral proximity mode mixing). The recent masking EMD method of Fosso and Molinas (2018) has shown the ability to solve the mode-mixing problem caused by components with spectral proximity (signal components that reside within the same octave). The simulated brain signals studied here exhibit these two types of mode-mixing problems concurrently, with the additional presence of dynamic variations (close spectral proximity and intermittency with dynamic transitions). Thus, we tested these two variants of EMD and the MEMD separately to assess their performance and limitations.

We have previously reported preliminary results on the use of EMD for this purpose, Bueno-Lopez et al. (2017a) and Bueno-Lopez et al. (2017b), but a comprehensive comparison with other pre-processing techniques in terms of the accuracy of reconstruction has not been available until now. Here, we make these main contributions:

1. A comprehensive analysis of the impact of the mode-mixing problem, inherent to the use of EMD, on the accuracy of brain-source spatio-temporal reconstruction
2. A comparison of the accuracy of spatio-temporal brain-source reconstruction using both EMD- and non-EMD-based techniques (WT)
3. A discussion concerning the impact of the type of brain signals on the choice of signal analysis tool to be used with MSP

We compared the accuracy of brain-source reconstruction using three EMD variants and wavelet-based analysis for the first time. This was for the purpose of discussing and proposing alternative solutions to the mode-mixing problem that EMD poses when applied to EEG signals in this specific area. From the EEG signals analyzed in this paper, we performed spatial localization (averaged over time) and spatio-temporal localization (for each time instant) of brain sources by decomposing the EEG signal into frequency bands. We compare and discuss the accuracy of the brain reconstruction, in terms of the Wasserstein metric (*w*_*m*_) using several methods (MSP, MSP with EMD, MSP with EEMD, MSP with masking EMD, MSP with multivariate EMD, and MSP with WPD): the lower the *w*_*m*_ value, the better the performed reconstruction. Our results show some of the limitations of spatio-temporal reconstruction; the optimal separation of modes is not possible by the separate use of EEMD and EMD with masking, due to the concurrent presence of close spectral proximity and intermittency in the studied signals. Although the mode-mixing problem has been largely reduced, mode mixing still persists as a consequence of the different types of mode-mixing targeted by EEMD and masking EMD and the simultaneous presence of different mode-mixing sources in the EEG signals. Despite these limitations, our results showed that the inclusion of a pre-processing stage based on EMD decomposition improves the performance of the MSP reconstruction method in terms of the spatial and temporal evolution of the neural activity.

## 2. Materials and methods

### 2.1. Empirical mode decomposition

EMD was proposed as an adaptive time-frequency data analysis method in Huang et al. (1998). EMD does not require any restrictive assumptions on the underlying model of the process/system under analysis and can accommodate both non-linear and non-stationary signals. However, the algorithm has been shown to have limitations in identifying closely-spaced spectral tones and intermittently appearing components in the signal (Bueno-Lopez et al., 2017b). The aim of the EMD method is to decompose the nonlinear and non-stationary signal *y*(*t*_*k*_) into a sum of IMFs that satisfies two conditions (Mandic et al., 2013):

1. Symmetric upper/lower envelopes (zero mean).
2. The number of zero-crossing and extrema that are either equal to or differ by exactly one.

The EMD algorithm for the signal *y*(*t*_*k*_) can be summarized as follows:

- Identify all extrema (maxima and minima) in *y*(*t*_*k*_).
- Interpolate between minima and maxima, generating the envelopes *e*_*l*_(*t*_*k*_) and *e*_*m*_(*t*_*k*_).
- Determine the local mean as *m*(*t*) = (*e*_*l*_(*t*_*k*_) + *e*_*m*_(*t*_*k*_))/2.
- Obtain the residue *r*(*t*_*k*_) = *y*(*t*_*k*_) − *m*(*t*_*k*_)
- Decide whether *r*(*t*_*k*_) is an IMF or not based on the two basic conditions for IMFs mentioned above.
- Repeat step 1 to 4 until *r*(*t*_*k*_) will be monotonic.

EMD is applied over ***y***(*t*_*k*_) to obtain ***γ***_*i*_(*t*_*k*_), *i* being the IMF, and

(1) $$\begin{array}{l} y\left(t_{k}\right)=\overset{N}{\underset{i=1}{\sum }}\gamma_{i}\left(t_{k}\right)+r\left(t_{k}\right) \end{array}$$

where *N* is the number of IMFs and ***r***(*t*_*k*_) a residual. Recently, several optimization techniques have been proposed to improve the performance of EMD (Hou and Shi, 2013; Xu et al., 2016).

Having obtained the intrinsic mode function components, we can apply the Hilbert transform to each component, and compute the instantaneous frequency according to Equation (2).

(2) $$\begin{array}{l} f_{i}\left(t\right)≜\frac{1}{2\pi }\cdot \frac{d\theta_{i}\left(t\right)}{dt}, \end{array}$$

where θ_*i*_(*t*) is the instantaneous phase of each IMF calculated from the associated analytic signal (Boashash, 1992). Finally, the instantaneous frequency can be observed in the Hilbert Spectrum.

### 2.2. Masking signal

The masking signal method reduces the problem of mode mixing for signals for which the components are close in frequency (e.g., when the frequencies of two adjacent IMFs are related by a factor ≤ 2). In this case, the EMD technique is unable to separate these components. The concept of a masking signal was first proposed by Deering and Kaiser (2005). The basic idea is to add a new signal to the analyzed signal that will prevent the mix of components with spectral proximity. Since the masking signal is known, it can be removed from the IMF through the EMD process which has been modified in the following way:

- Construct a masking signal *s*(*t*_*k*_), from the frequency information of the original data, *y*(*t*_*k*_).
- Perform EMD on *y*_+_(*t*_*k*_) = *y*(*t*_*k*_) + *s*(*t*_*k*_) to obtain the IMF *z*_+_(*t*_*k*_). Similarly obtain *z*_−_(*t*_*k*_) from *y*_−_(*t*_*k*_) = *y*(*t*_*k*_) − *s*(*t*_*k*_).
- Define the IMF as *z*(*t*_*k*_) = (*z*_+_(*t*_*k*_) + *z*_−_(*t*_*k*_))/2.

The difficulty with this method is choosing the amplitude and frequency of the masking signal *s*(*t*_*k*_). According to Deering and Kaiser (2005), a good choice results in each frequency within the signal being separated by at least a factor of 2, is *s*(*t*_*k*_) = *a*_0_sin(2π*f*_*s*_*t*). Although some general indications are provided on how to choose *a*_0_ and *f*_*s*_, the reported process is mostly empirical and user experience is required for the selection of the signal parameters for a particular problem. A new masking-signal method has been recently developed by Fosso and Molinas (2018) to separate spectral components that reside within the same octave. In contrast to the guidelines presented by Deering and Kaiser (2005) for selecting the amplitude and frequency of the masking signal, this work defines precise amplitudes and frequencies to reverse a mode-mixing condition using a boundary map built for a composite of two-signals across the entire frequency spectrum. The masking EMD method implemented here is based on this work and the frequencies and amplitudes of the masking signal are selected according to the boundary map presented in Fosso and Molinas (2018). The procedure for removing the masking signal from the set of IMFs is identical to that described above (Deering and Kaiser, 2005).

### 2.3. Ensemble empirical mode decomposition

Wu and Huang proposed a noise-assisted data analysis (NADA) method, ensemble empirical mode decomposition (EEMD), which defines the true IMF components as the mean of an ensemble of trials, each consisting of the signal plus white noise of finite amplitude (Wu and Huang, 2009). EEMD is carried out as follows:

- Add a white noise series to the data base *y*(*t*_*k*_).
- Decompose the data with added white noise using EMD to obtain the IMFs.
- Repeat steps 1 and 2 again, but with a different white noise series each time.
- Obtain the (ensemble) mean of the corresponding IMFs of the decomposition as the final result.

The main effect of decomposition using EEMD is that the added white noise series cancel each other in the final mean of the corresponding IMFs. Modified versions of the EEMD have been recently proposed. Torres et al. (2011), proposed a variation of EEMD, complete ensemble empirical mode decomposition with adaptive noise (CEEMDAN) and an improved version of CEEMDAN can be found in Colominas et al. (2014).

### 2.4. Multivariate EMD

Three issues arise when univariate EMD is applied (channel by channel) to multichannel signals, namely: *the nonuniformity, scale alignment and nature of IMFs*, which are a problem in data/image fusion applications (Mandic et al., 2013). The local maxima and minima of multivariate signals cannot be directly defined and the notion of “oscillatory modes” to define an IMF is confusing in this case (Rehman and Mandic, 2010). This method proposes taking signal projections in multiple directions that have been distributed in a uniform way within an n-dimensional space to obtain multiple envelopes which are averaged and then interpolating (using a cubic spline) their extrema to estimate the local n-dimensional mean. Special attention is required to choose a suitable set of directions from the signal projections taken in the n-dimensional space (Mandic et al., 2013).

The following algorithm summarizes how MEMD works:

1. Using the Hammersley sequence, as a uniformly sampling a n-dimensional sphere, generate a P-point.
2. Projections *q*_θ_*p*__(*t*_*k*_) of the signal *y*(*t*_*k*_) must be calculated in the same direction vector **x**_θ_*p*__, for *p* = 1, …, *P* and then to obtain a set of projections {*q*_θ_*p*__(*t*_*k*_)}${\;}_{p=1}^{P}$
3. Find the instants in time {$t_{\theta_{p}}^{i}$}${\;}_{p=1}^{P}$ that correspond to the maxima of the set of projections of the signals {*q*_θ_*p*__(*t*_*k*_)}${\;}_{p=1}^{P}$
4. Interpolate [$t_{\theta_{p}}^{i}$, *s*($t_{\theta_{p}}^{i}$)] to obtain the envelope curves {*e*_θ_*p*__(*t*_*k*_)}${\;}_{p=1}^{P}$
5. Calculate the mean of the P multidimensional envelopes
  (3) $$\begin{array}{l} m\left(t_{k}\right)=\frac{1}{P}\overset{P}{\underset{p=1}{\sum }}e_{\theta_{p}}\left(t_{k}\right) \end{array}$$
6. Extract the “detail" *d*(*t*_*k*_) = *s*(*t*_*k*_) − *m*(*t*_*k*_). If *d*(*t*_*k*_) fulfills the stoppage criterion for a multivariate IMF, apply the above procedure to *s*(*t*_*k*_) − *d*(*t*_*k*_), otherwise, repeat *d*(*t*_*k*_).

### 2.5. Multi-signal wavelet packet decomposition (WPD)

This method allows projection of the multi-channel EEG signals into several sub-spaces *V*_(*j, i*)_, *j* = 0, …, *j* being the number of decomposition levels, and *i* = 0, …, 2^*j*^ − 1 the number of sub-bands of each level. The decomposition must satisfy:

(4) $$\begin{array}{l} V_{\left(j,i\right)}=V_{\left(j+1,2^{i}\right)}⊕V_{\left(j+1,2^{i}+1\right)} \end{array}$$

A subset of sub-spaces can be selected to reconstruct the original signal using a cost function e.g., an entropy-based cost function or retained energy.

Multi-signal WPD creates two possibilities to rebuild brain activity: the first is to reconstruct the brain activity from each sub-band, which allows sub-band brain mapping. The second is spatial brain activity reconstruction obtained from a combination of the sub-spaces that retain the most relevant information e.g., an entropy-based cost function (Muñoz-Gutiérrez et al., 2018). However, the dyadic reduction of time resolution inherent to wavelet decomposition must be considered as a constraint if a spatio-temporal reconstruction is required.

### 2.6. EEG source localization: the neuromagnetic inverse problem

EEG recordings are obtained from a limited number of sensors, whereas the number of possible active sources in the brain is close to 2,000. The consequence is an ill-posed and mathematically undetermined problem which must be solved. This problem, called the neuromagnetic inverse problem, can be solved using prior information (spatial, temporal, or frequency) related to neuronal activity, either based on the geometric or physiological properties of the brain, which can be included as constrains. These suitable constrains allow a unique approximate solution to the inverse problem (Babadi et al., 2014; Giraldo-Suarez et al., 2016). Over the last 25 years, various approaches have been proposed to solve the neuromagnetic inverse problem from regularization methods [minimum norm estimated (MNE), low resolution tomography (LORETA), iterative regularization algorithms (IRA), time-frequency mixed norm estimate (TF-MxNE), and spatio-temporal unifying tomography (STOUT)] to Bayesian frameworks (MSP, hierarchical Bayes model), and some approaches with spatio-temporal constraints that also include frequency information (Friston et al., 2008; Gramfort et al., 2013; Babadi et al., 2014; Castaño-Candamil et al., 2015; Giraldo-Suarez et al., 2016; Costa et al., 2017). The dynamic behavior of the brain can be accounted for using a dynamic model as a constraint for neural activity estimation. Many approaches can be used, each with different constrains or models to solve the inverse problem of brain activity reconstruction to obtain the information on the active sources (Babadi et al., 2014).

In general, we must consider a model of EEG generation from known neural activity given by

(5) $$\begin{array}{l} y\left(t_{k}\right)=Mx\left(t_{k}\right)+\epsilon \left(t_{k}\right) \end{array}$$

with $y\left(t_{k}\right)\in {\mathbb{R}}^{d\times 1}$ being the EEG at sample time *t*_*k*_, ***M*** ∈ ℝ^*d*×*n*^ the leadfield matrix, and $x\left(t_{k}\right)\in {\mathbb{R}}^{n\times 1}$ the neural activity. According to (5) the inverse problem can be defined as the estimation of neural activity ***x***(*t*_*k*_) based on the EEG measurements ***y***(*t*_*k*_) and the knowledge of the leadfield matrix ***M***. Additional information can be considered in the solution of the inverse problem by considering the inherent spatio-temporal dynamics of EEG signals, resulting in a dynamic inverse problem solution. Here, we performed neural activity reconstruction using the MSP method, proposed by Friston et al. (2008). The MSP method considers spatio-temporal constraints based on application of the hierarchical or empirical Bayes model to the distributed source reconstruction problem in EEG, in which an automatic selection of multiple cortical sources with compact spatial support are specified in terms of empirical priors.

Here, we did not directly perform the brain activity reconstruction over the EEG ***y***(*t*_*k*_), but computed the EEG source localization over each resulting IMF ***γ***_*i*_(*t*_*k*_) obtained from (1), as proposed by Bueno-Lopez et al. (2017a). We obtained the IMFs by applying the aforementioned methods: EMD, EMD with masking signal, EEMD, Multivariate EMD and Wavelet Packets decomposition.

## 3. Data analysis

### 3.1. Experimental setup

The central idea was to show how EMD and its variants can be used for the reconstruction of brain activity from EEG signals. Access to a standard EEG database is important because it is necessary to know the underlying source activity to evaluate the methods for solving the inverse problem. We used a model with *n* = 20, 484 sources and 30 electrodes for simulation, as described by Giraldo-Suarez et al. (2016).

With this head model the neuronal activity ***x***(*t*_*k*_) is obtained by considering windowed sinusoidal signals with a sampling rate of 100 Hz. In this case, three sources randomly located into the brain are selected, for which the activity in each source is generated according to the following expression:

(6) $$\begin{array}{l} x_{i}\left(t_{k}\right)=e^{-\frac{1}{2}{\left(\frac{t_{k}-c_{i}}{\sigma }\right)}^{2}}\sin\left(2\pi f_{i}t_{k}\right), \end{array}$$

**Figure 1:**
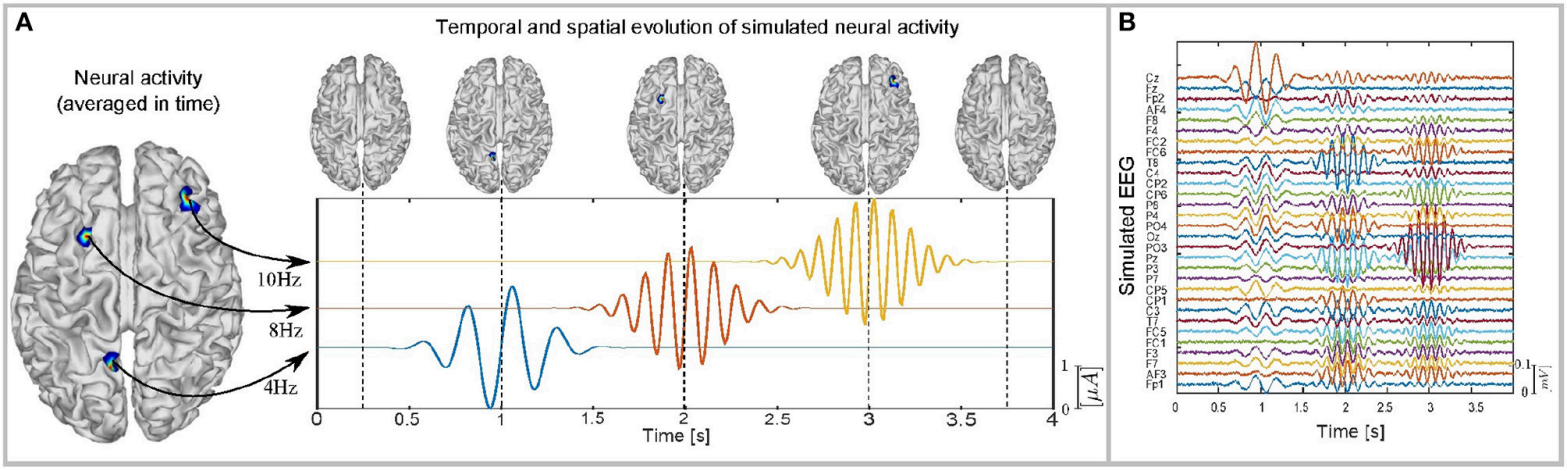
**(A)** Temporal and spatial evolution of Simulated Neural Activity an its corresponding brain mapping for each time instant and averaged in time. **(B)** Simulated EEG for an SNR of 10 dB.

*c*_*i*_ being the center of the windowed signal in seconds (1, 2 and 3 s), and *f*_*i*_ the frequency of the signal (4, 8, and 10 Hz), with *i* = 1, 2, 3 and σ = 0.2. The windowed activity is selected to obtain EEG signals that contain specific frequencies for only finite times, which facilitates the performance analysis of the pre-processing methods being tested. The EEG is simulated according to (5), where a Signal-to-Noise-Ratio (SNR) of 10 dB is considered, according to Bueno-Lopez et al. (2017a). The simulated neural activity, with its temporal and spatial evolution, is shown in Figure 1A, and the simulated EEG for an SNR of 10dB is shown in Figure 1B. The source activity behavior described in (6) can be seen in real EEG signals, such as evoked potentials resulting from visual stimulus or mental imagery (Henson and Wakeman, 2015; Giraldo-Suarez et al., 2016) or focal epilepsy seizures (Martinez-Vargas et al., 2017), for at least one active source at each instance of time.

The frequencies were chosen within the theta-alpha frequency bands because, according to Agyei et al. (2016), some signs of infant immaturity have been associated with low-frequency rhythms in infants and activities over visual areas exhibit low-amplitude de-synchronization when motion stimuli were compared with static stimuli. In addition, some studies on epilepsy, e.g., that of Martinez-Vargas et al. (2017), have supported their preliminary results modeling seizure activity through sinus function, for which the frequency varied smoothly from 12 to 8 Hz. Moreover, the signals that we studied exhibited a combination of concurrent spectral proximity mode mixing and intermittency mode mixing, which is challenging for the EMD methods discussed in this work, when used separately.

The MSP method for neural activity reconstruction was applied to the resulting IMFs or directly to the raw EEG data to evaluate the quality of the neural activity reconstruction when EMD, EEMD, masking EMD, multivariate EMD, or WPD were applied

The neural activity reconstruction was analyzed by considering the temporal and spatial evolution of the underlying simulated activity, as well as the neural activity averaged over time. In addition, the Wasserstein metric *w*_*m*_ was used as a performance index to obtain a quantitative comparison of the quality of the reconstructed neural activity (Lucka et al., 2012). The index *w*_*m*_ measures the effort needed to transform the estimated power distribution into the actual distribution by transporting the probability mass (Castaño-Candamil et al., 2015). Thus, the lower the *w*_*m*_ value, the better the performance of the estimated neural activity reconstruction.

**Figure 2:**
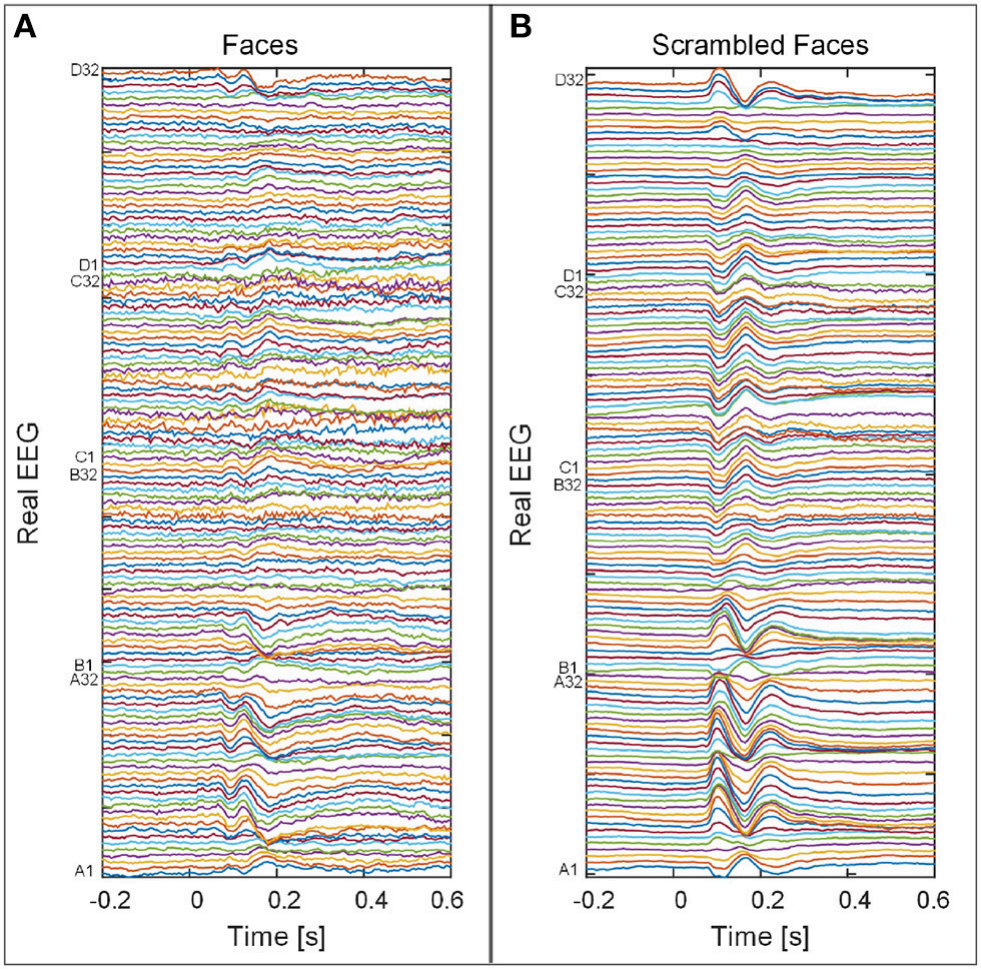
Real signals corresponding to 128 EEG channels for two cases: **(A)** Subjects exposed to faces. **(B)** Those exposed to scrambled faces.

The set of real signals contains evoked potentials acquired from a subject who gave written consent to participate in a multi-modal study of face perception. The data were recorded while making symmetry judgments of faces and scrambled faces, as described in Henson and Wakeman (2015). Faces were presented for 600 ms, every 3, 600 ms while data were acquired on a 128-channel ActiveTwo system, sampled at 2, 048 Hz. After artifact rejection, the epochs were baseline-corrected from -200 to 0 ms, averaged over each condition and down-sampled to 200 Hz, as depicted in Figure 2. The source space was modeled using a tessellated surface in the gray-white matter interface with *n* = 8, 196 vertices with source orientations fixed orthogonally to the surface. Also, the lead fields were computed using the BEM volume conductor model, with the mean distance between neighboring vertices adjusted to 5 mm. The Wasserstein metric was also used to evaluate the quality of the spatial reconstruction (averaged over time) by assuming that the Functional magnetic resonance imaging (fMRI) reconstruction was the ground truth.

### 3.2. Results and analysis

**Figure 3:**
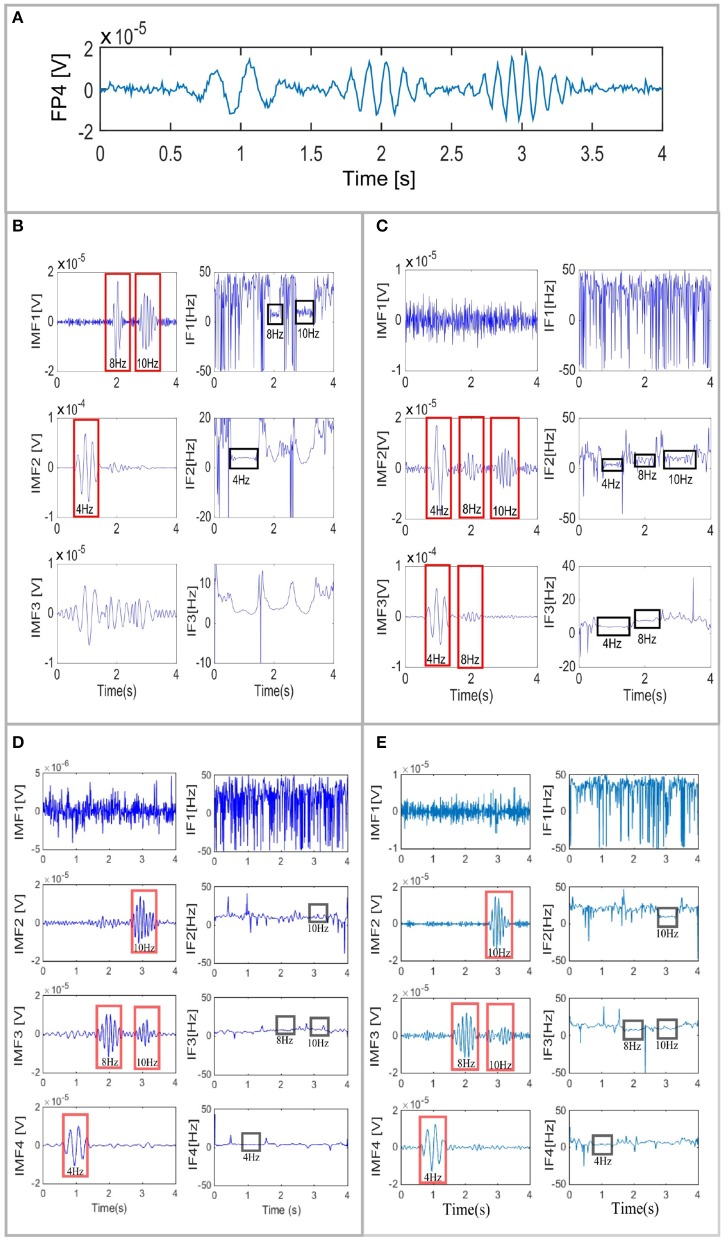
EEG signal of channel FP4 **(A)** and its corresponding IMF decomposition and IFs using EMD **(B)**, EEMD **(C)**, masking EMD **(D)**, and multivariate EMD **(E)**. Mode mixing and mode splitting are observed in all cases but they are reduced in cases **(D,E)**.

The IMFs obtained for one (channel Fp4 as shown in Figure 3A) of the 30 using EMD (Huang et al., 1998), EEMD (Wu and Huang, 2009), masking EMD from Fosso and Molinas (2018), and multivariate EMD from Mandic et al. (2013) are shown in Figures 3B–E, respectively. The FP4 channel was selected to exemplify the EMD decomposition and its variants, as this channel exhibits all the amplitudes of the underlying neural activity with the same amplitude. Channel FP4 is shown at the top of Figure 3A. EMD (Figure 3B) showed clear mode mixing. Simple inspection of the instantaneous frequency revealed two frequency components (8 Hz in *t* = 2 s, and 10 Hz in *t* = 3 s) and the noise in the first IMF. The other two IMFs show the 4 Hz source in *t* = 1 s but in general they provided limited information, as the instantaneous frequency was strongly fluctuating. When EEMD was applied for decomposition, all the information of interest was contained in the second and third IMFs (Figure 3C), whereas the additive noise appeared in the first IMF.

By using the EMD with the same masking signal for all the channels simultaneously, as follows:

(7) $$\begin{array}{l} s\left(t_{k}\right)=3\sin\left(2\pi 29t_{k}\right)+\sin\left(2\pi 14.5t_{k}\right)+\sin\left(2\pi 8t_{k}\right)\\ \;+0.7\sin\left(2\pi 4t_{k}\right), \end{array}$$

it is possible to more clearly distinguish the noise and the three different frequencies that appear Figure 3D. In Figure 3D, the first IMF contains the additive noise, whereas the second, third, and fourth IMFs, contain the activity in the frequencies of 10, 8, and 4 Hz, respectively. A small part of the brain activity in the 10-Hz range activity in *t* = 3 s appears also in IMF3. However, even when mode mixing persists in the third IMF of Figure 3D, it is possible to clearly identify the instant in which each component appears in each IMF. Moreover, multivariate EMD (Figure 3E) behaved like masking EMD (Figure 3D), but the fluctuations of the instantaneous frequency in the IMFs resulting from MEMD resulted in some noise in the second and third IMFs.

**Figure 4:**
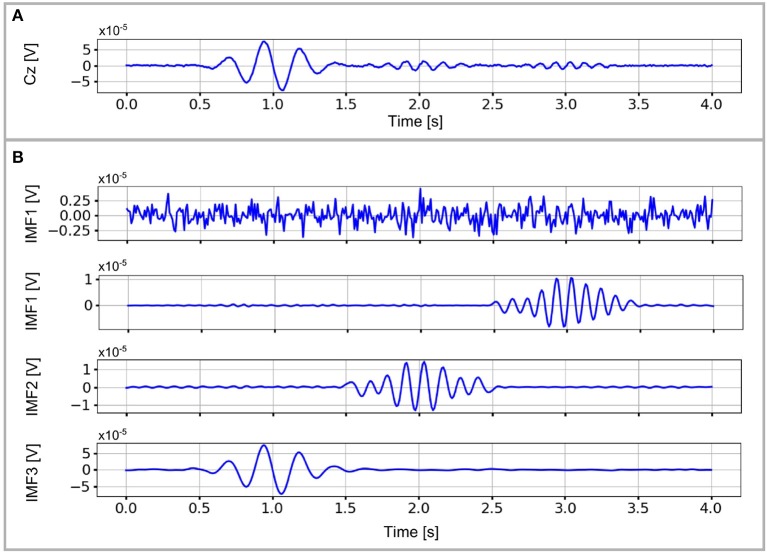
EEG signal of the Cz channel **(A)** and its corresponding IMF decomposition using a masking signal with variable amplitude and frequency **(B)**. The three neural activity sources and the noise are clearly separated each in a single IMF with no mode mixing.

An example of the performance that can be obtained with masking EMD is presented in Figure 4. The EEG signal of the Cz channel is shown in Figure 4A, and its corresponding IMFs decomposition in Figure 4B, in which perfect separation of neural activity was obtained (no mode mixing or mode splitting).

The masking signals used in this example consider variable amplitude and frequency and are defined as follows:

(8) $$s_{1}(t_{k})=\sin(2\pi 20t_{k})$$

(9) $$s_{2}(t_{k})=a_{1}(t_{k})\sin(2\pi f_{1}(t_{k})t_{k})+s_{1}(t_{k})$$

(10) $$s_{3}(t_{k})=a_{2}(t_{k})\sin(2\pi f_{2}(t_{k})t_{k})+s_{1}(t_{k})$$

(11) $$s_{4}(t_{k})=0.7\sin(2\pi 4t_{k})$$

with *a*_*i*_(*t*_*k*_) and *f*_*i*_(*t*_*k*_) defined as follows:

$$\begin{array}{ll} a_{1}(t_{k})=\{\begin{array}{l} 0.5;0\leq t_{k}\leq 2.5\\ 1.5;2.5\leq t_{k}\leq 3.5\\ 0.7;3.5\leq t_{k}\leq 4 \end{array} & f_{1}(t_{k})=\{\begin{array}{l} 11;0\leq t_{k}\leq 2.5\\ 10;2.5\leq t_{k}\leq 3.5\\ 11;3.5\leq t_{k}\leq 4 \end{array}\\ a_{2}(t_{k})=\{\begin{array}{l} 0.5;0\leq t_{k}\leq 1.5\\ 2;1.5\leq t_{k}\leq 2.5\\ 0.5;2.5\leq t_{k}\leq 4 \end{array} & f_{2}(t_{k})=\{\begin{array}{l} 10;0\leq t_{k}\leq 1.5\\ 8;1.5\leq t_{k}\leq 2.5\\ 10;2.5\leq t_{k}\leq 4 \end{array} \end{array}$$

The masking signals *s*_*i*_(*t*_*k*_), for *i* = 1, …, 4 are applied sequentially. In addition, the application of masking EMD resulted in no mode mixing or mode splitting in the resulting decomposition (Figure 4). However, the masking signal required to obtain such performance, as shown in (8), is complex and channel dependent. Thus, we limited the selection of the masking signal for masking EMD to one signal for all analyzed channels.

**Figure 5:**
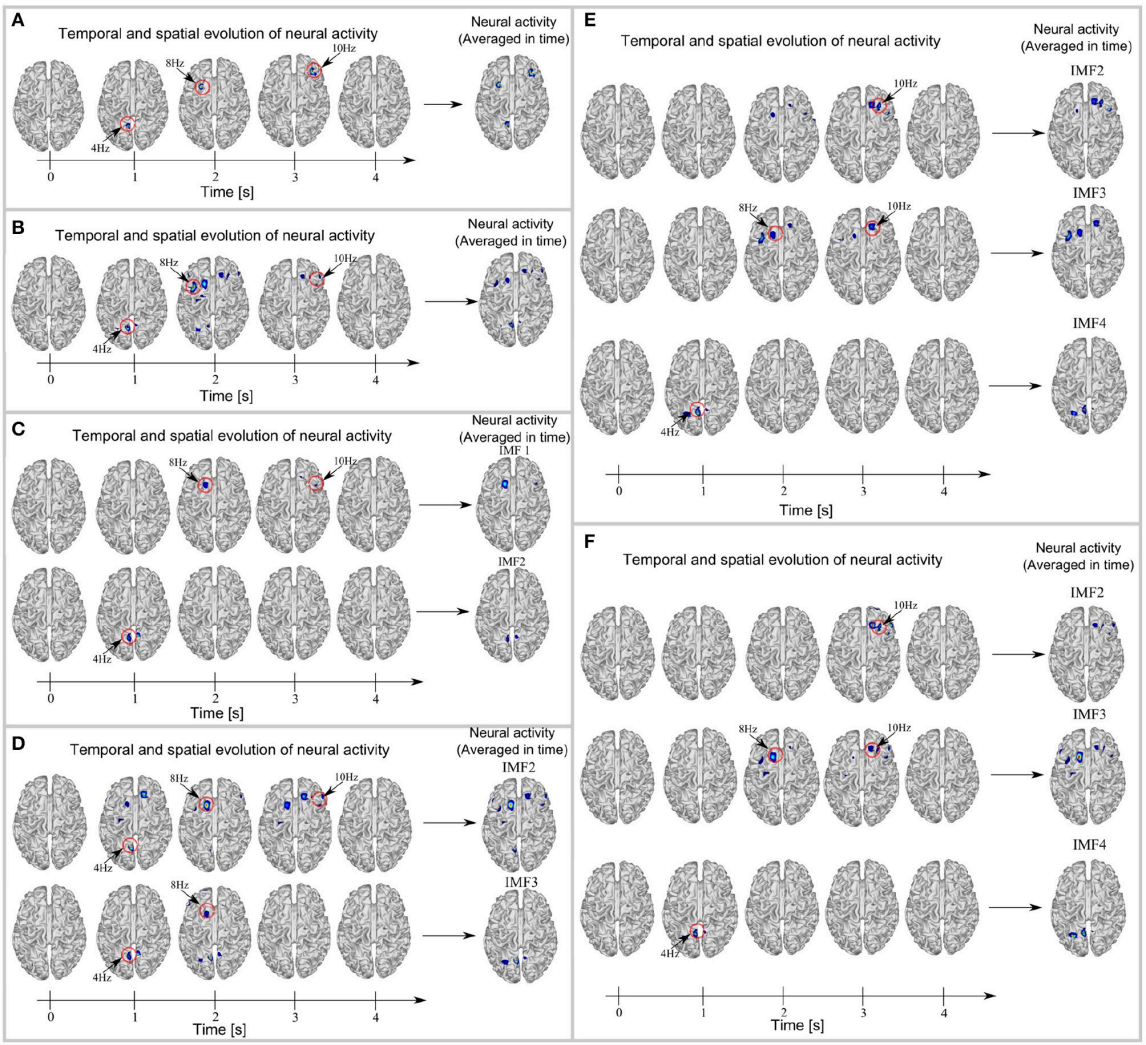
Original brain activity or ground truth **(A)**, brain reconstruction based on raw EEG signals using MSP **(B)**, and MSP-based mapping of IMF's resulting from EMD **(C)**, EEMD **(D)**, masking EMD **(E)**, and multivariate EMD **(F)**. For each case the temporal and spatial evolution is shown. At *t* = 1*s* occurs the 4 Hz activity, at *t* = 3*s* and *t* = 5*s* occur the 8 and 10 Hz activities respectively. The spurious activity observed in several spatial reconstructions represent either the mode mixing or mode splitting phenomena.

**Figure 6:**
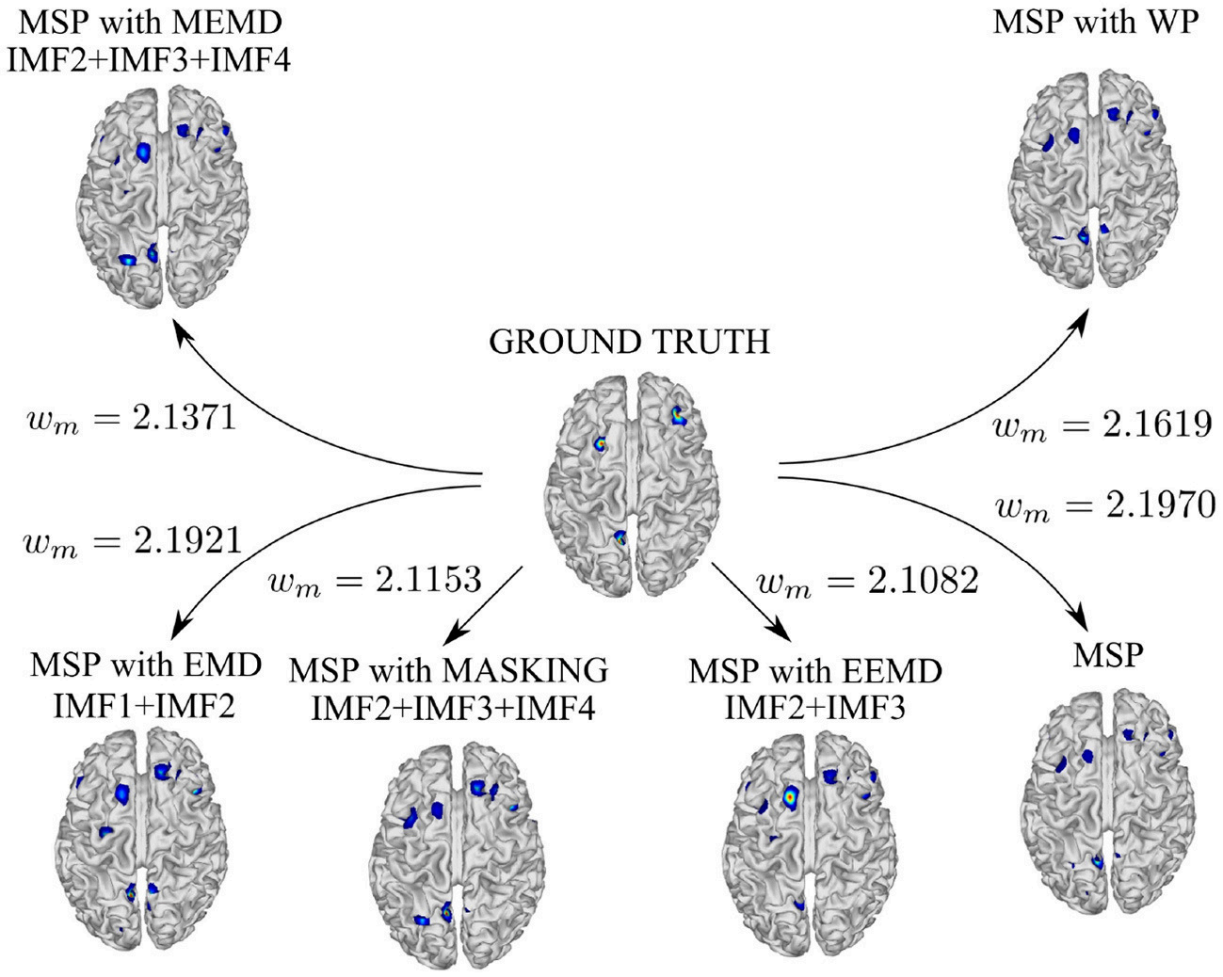
Reconstruction performance in terms of the Wasserstein metric for simulated signals.The best reconstructions were obtained using masking EMD (*w*_*m*_ = 2.1153) and EEMD (*w*_*m*_ = 2.1082), that yielded the lowest Wasserstein metric values.

Figure 5 shows the temporal and spatial evolution of brain activity (during five instants in time) and its average over time for the following cases: ground truth (Figure 5A), MSP with raw data (Figure 5B), MSP with EMD (Figure 5C), MSP with EEMD (Figure 5D), MSP with EMD using a masking signal (Figure 5E), and MSP with multivariate EMD (Figure 5F). In addition, the reconstruction performance in terms of the Wasserstein metric is shown in Figure 6.

Figure 5A shows the spatial and temporal evolution of ground truth neural activity and its average over time, in which the windowed activity of the source at 4 Hz appears at time *t* = 1 second, and the windowed activity of the sources at 8 and 10 Hz appears at *t* = 2 and *t* = 3 s, respectively. The full reconstruction of brain activity using MSP is shown in Figure 5B. The partial reconstruction of the brain activity using MSP with EMD from data obtained from IMF 1 (at the top) and IMF 2 (at the bottom) are shown in Figure 5C. Of note, the two IMFs were obtained after applying EMD to the simulated EEG signals.

Figures 5D–F show the partial brain reconstructions obtained after applying MSP to IMFs obtained from EEMD (IMF 2 and IMF 3), masking EMD (IMF 2, IMF 3, and IMF 4), and multivariate EMD (IMF 2, IMF 3, and IMF 4), respectively. The temporal and spatial evolution of neural activity shown for each model in Figure 5, is consistent with the temporal evolution of the brain activity and the temporal behavior of the IMFs presented in Figures 1, 3.

The reconstruction performance in terms of the Wasserstein metric is presented in Figure 6. According to the Wasserstein metric, the best approximations were obtained using masking EMD (Figure 5E) and EEMD (Figure 5D). In both cases, the three components were identified in different IMFs: (IMF 2, IMF 3, and IMF 4 for masking EMD and IMF 2 and IMF 3 for EEMD). These reconstructions outperformed those obtained using multivariate EMD (Figure 5F) or Wavelet Packet Decomposition. This was due to the fact that the fluctuation in instantaneous frequency in masking EMD and EEMD were smaller than when the other methods were used.

**Figure 7:**
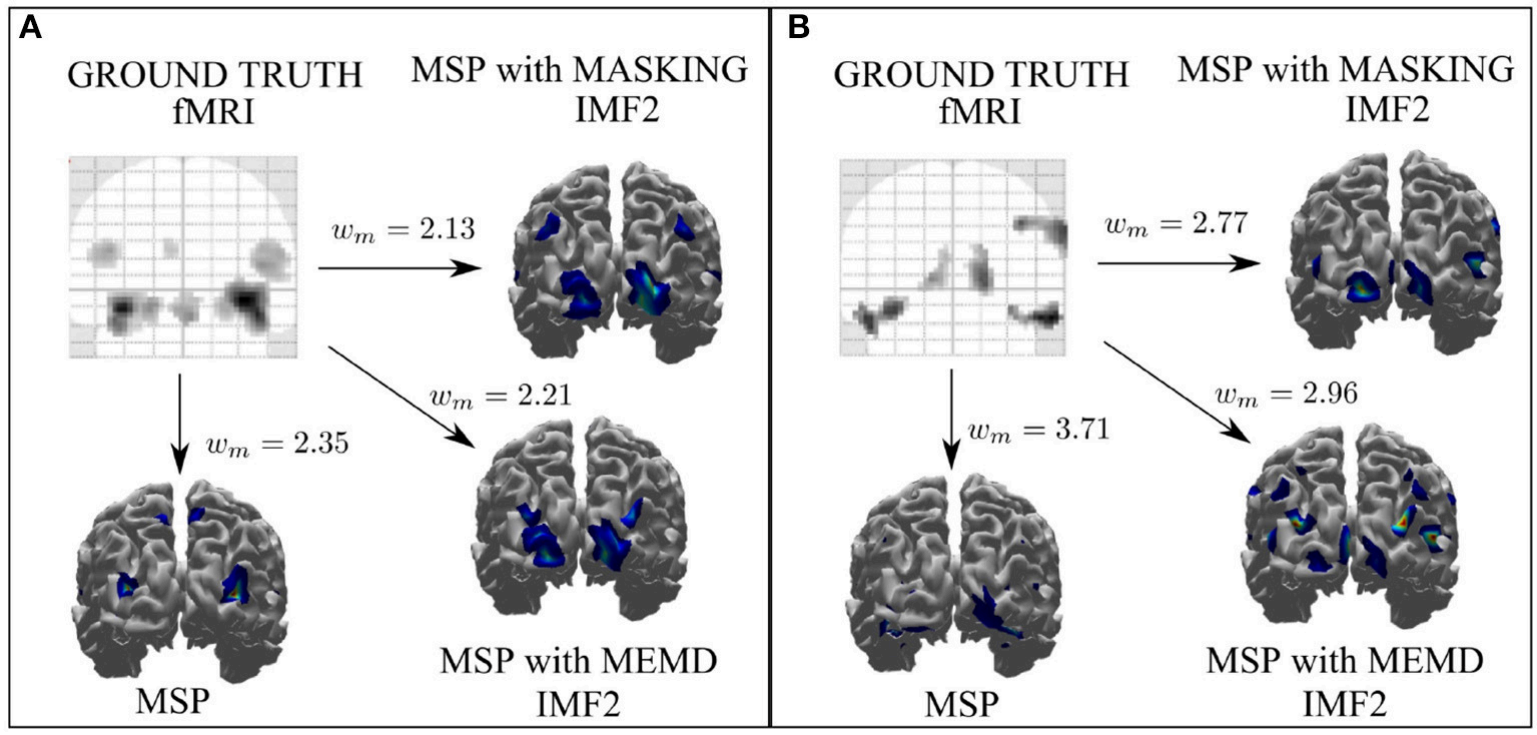
Reconstruction performance of MSP, MEMD, and masking EMD, vs. ground truth (fMRI) in terms of the Wasserstein metric for real EEG signals for two cases: subjects exposed to faces **(A)** and those exposed to scrambled faces **(B)**. The best reconstruction in cases **(A,B)** were obtained using masking EMD with *w*_*m*_ = 2.13 and *w*_*m*_ = 2.77, respectively, which yielded the lowest Wasserstein metric values.

We performed a similar analysis for the spatial reconstruction achieved from real EEG signals using MSP with raw data, multivariate EMD, and masking EMD. The reconstruction performance in terms of the Wasserstein metric is presented in Figure 7 for two cases: subjects exposed to faces and those exposed to scrambled faces. According to the Wasserstein metric, the best approximations were obtained using masking EMD for both cases (subjects exposed to faces and scrambled faces), where the following masking signal was used *s*(*t*_*k*_) = 3sin(2π8*t*_*k*_).

**Figure 8:**
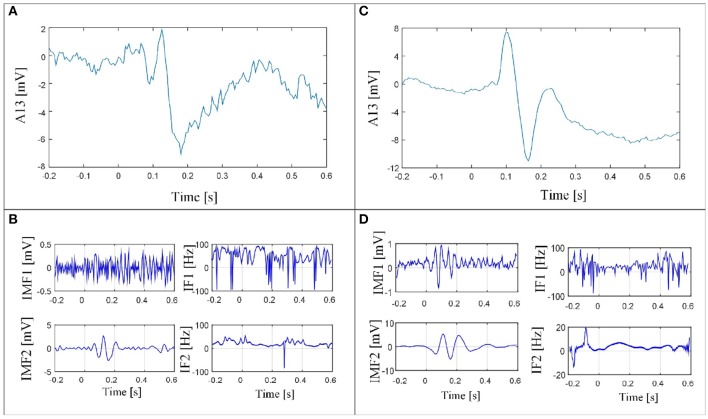
EEG signal of channel A13 for subjects exposed to faces **(A)** and its corresponding IMF decomposition using masking EMD **(B)**. EEG signal of channel A13 for subjects exposed to scrambled faces **(C)** and its corresponding IMF decomposition using masking EMD **(D)**.

Figure 8 shows the IMF decomposition for the masking-EMD method and their corresponding IF for channel A13 for two cases: the faces paradigm (Figures 8A,B) and the scrambled faces paradigm (Figures 8C,D). These were the results that yielded the lowest Wasserstein metric value (Figure 7). The noise and activity related to the evoked potential recorded in channel A13 were also adequately unmixed. In addition, the evoked potentials for IMF2 (Figures 8B,D) were localized in time (windowed) and exhibited similar behavior to the simulated signals.

## 4. Discussion and conclusions

This study investigated the combined use of EMD-MSP analysis for EEG data to localize sources of neural activity in the brain. The analysis has been performed in three different ways: (i) using the raw data of the sources of brain activity, (ii) using three EMD variants (EEMD, MEMD, and masking EMD) in addition to the standard EMD and (iii) using the WT pre-processing to solve the inverse problem with MSP. The study showed that the spatio-temporal reconstruction of brain-source activity can be severely affected by the mode-mixing problem when using EMD as a pre-processing tool with MSP to identify the location of brain activity. We implemented three versions of an EMD solution to cope with the mode-mixing problem and tested them in simulated and real EEG signals in an attempt to improve brain activity reconstruction when using EMD. We tested masking EMD, EEMD, and MEMD, which have been shown to be effective in other fields, for pre-processing the raw EEG signals before implementing MSP for brain source reconstruction. We also implemented pre-processing based on the wavelet transform to compare spatio-temporal reconstruction based on this method with those based on EMD and the ground truth. The sub-band reconstruction effectively split the brain activity into frequency bands (Figure 5). Visual inspection shows that the noise is adaptively filtered in one IMF (IMF1, as shown in Figure 3B), particularly for the EEMD method, improving the neural activity reconstruction, which is computed using other IMFs (IMF2 and IMF3, as shown in Figure 5E). However, this method showed a drawback in the reconstructions obtained at low frequencies, as the activity was scattered throughout several IMFs. A salient property of EMD that emerges from this study is that each of the bands represented by a single IMF can be associated with a source of brain activity when effective EMD separation is achieved (e.g., when the mode-mixing problem is minimized). This can be very useful for accurate functional brain reconstruction using EEG. In addition, the IF of each IMF (shown in Figure 3) can be very useful for the detection of instantaneous variations in the frequencies of each band when mode mixing is reduced to a minimum.

For all the cases we studied, the accuracy of the spatio-temporal reconstruction was quantified using the Wasserstein metric for the reconstruction averaged over time, but it can be evaluated for each instant in time as well. The best spatial reconstructions were achieved with EEMD and masking EMD. If we compare our results to the ones obtained with combined EMD-sLORETA approach in Karema et al. (2016) for the same signals used in our paper, the reconstruction accuracy we obtained with EEMD-MSP is slightly better than the EEMD-sLORETA in Karema et al. (2016). The difference is small, and it can be attributed to the different brain mapping methods used in the two studies. When WT is used for decomposition, our results compared to the approach in Muñoz-Gutiérrez et al. (2018) for the same signals, gives the same accuracy when using the WT for signal decomposition. This is well in line with our results using wavelet packets. When compared to the WT decomposition presented in Korats et al. (2016) their results are comparable with our results when using WT decomposition (wavelet packets). However, their approach requires very long signal segments for the process of optimization and a-priory knowledge of the number of sources. These comparisons verify the general trend observed in our results, that the EMD-based methods obtained a better spatio-temporal reconstruction.

As far as mode mixing is concerned, when we compare our results using masking EMD to the methodology presented in Deering and Kaiser (2005), we obtained reduced mode mixing and better reconstruction of neuronal activity compared to the one presented in Bueno-Lopez et al. (2017a) which is based on the masking in Deering and Kaiser (2005). In Karema et al. (2016), the use of EEMD-sLORETA is justified by the fact that mode mixing was attributed only to intermittency and the results were not compared with other variants of EMD. In our study, both masking EMD and EEMD achieved a good temporal reconstruction that identified the three active sources, but the spatial reconstruction (when the temporal and spatial evolution of neural activity was analyzed) still suffered from the mode-mixing problem. This is partly due to the fact that EEMD and masking EMD target different types of mode-mixing problems, while the signals studied here exhibited both types of mode mixing contemporaneously. Indeed, the mode-mixing type is signal-dependent and the optimal pre-processing tool to apply to a given signal can be a combination of more than one principle. Here, EEMD and masking EMD were applied separately to better understand their properties associated with the studied signals. Another reason for the remaining mode mixing is that the masking signal implemented in this study was the same for all channels, whereas the most favorable masking signal always depends on the single signal and its mode mixing attributes. We are currently developing a generalized algorithm that will optimally combine the strengths of both EEMD and masking EMD aimed at addressing these two types of mode-mixing problems when they occur contemporaneously.

The results of this study, which focuses on accurately localizing sources of neural activity through inverse modeling of EEG data treated with EMD, matched well with the ground truth. From a review of the performance of previous approaches to EEG decomposition, one could conclude that the combined use of EMD-MSP analysis gives satisfactory results. In general, EEMD and masking EMD gives better results than WT, not only because of a better temporal resolution, but because the separation in frequency bands is adaptive. These results may be relevant in localizing the source of an epileptic seizure and in the detection of seizures.

## Author contributions

PM-G, EG, and MB-L conceived, designed, and performed the experiments. MM suggested the use of EMD, described the design principle of masking EMD, interpreted the results, and analyzed the data. All authors discussed the results and wrote and refined the article.

### Conflict of interest statement

The authors declare that the research was conducted in the absence of any commercial or financial relationships that could be construed as a potential conflict of interest.
